# Computational reinforcement learning, reward (and punishment), and dopamine in psychiatric disorders

**DOI:** 10.3389/fpsyt.2022.886297

**Published:** 2022-10-20

**Authors:** Brittany Liebenow, Rachel Jones, Emily DiMarco, Jonathan D. Trattner, Joseph Humphries, L. Paul Sands, Kasey P. Spry, Christina K. Johnson, Evelyn B. Farkas, Angela Jiang, Kenneth T. Kishida

**Affiliations:** ^1^Neuroscience Graduate Program, Wake Forest University School of Medicine, Winston-Salem, NC, United States; ^2^Department of Physiology and Pharmacology, Wake Forest University School of Medicine, Winston-Salem, NC, United States; ^3^Georgia State University Undergraduate Neuroscience Institute, Atlanta, GA, United States; ^4^Department of Neurosurgery, Wake Forest University School of Medicine, Winston-Salem, NC, United States; ^5^Department of Biomedical Engineering, Wake Forest University School of Medicine, Winston-Salem, NC, United States

**Keywords:** punishment learning, reward learning, reward prediction error (RPE), temporal difference (TD) learning, depression, addiction

## Abstract

In the DSM-5, psychiatric diagnoses are made based on self-reported symptoms and clinician-identified signs. Though helpful in choosing potential interventions based on the available regimens, this conceptualization of psychiatric diseases can limit basic science investigation into their underlying causes. The reward prediction error (RPE) hypothesis of dopamine neuron function posits that phasic dopamine signals encode the difference between the rewards a person expects and experiences. The computational framework from which this hypothesis was derived, temporal difference reinforcement learning (TDRL), is largely focused on reward processing rather than punishment learning. Many psychiatric disorders are characterized by aberrant behaviors, expectations, reward processing, and hypothesized dopaminergic signaling, but also characterized by suffering and the inability to change one's behavior despite negative consequences. In this review, we provide an overview of the RPE theory of phasic dopamine neuron activity and review the gains that have been made through the use of computational reinforcement learning theory as a framework for understanding changes in reward processing. The relative dearth of explicit accounts of punishment learning in computational reinforcement learning theory and its application in neuroscience is highlighted as a significant gap in current computational psychiatric research. Four disorders comprise the main focus of this review: two disorders of traditionally hypothesized hyperdopaminergic function, addiction and schizophrenia, followed by two disorders of traditionally hypothesized hypodopaminergic function, depression and post-traumatic stress disorder (PTSD). Insights gained from a reward processing based reinforcement learning framework about underlying dopaminergic mechanisms and the role of punishment learning (when available) are explored in each disorder. Concluding remarks focus on the future directions required to characterize neuropsychiatric disorders with a hypothesized cause of underlying dopaminergic transmission.

## Introduction

Psychiatric conditions diagnosed within DSM-5 (1) criteria are based on clinical phenomenon (e.g., patient-reported symptoms and clinician-observation signs) rather than quantifiable physiological markers. Therapeutic regimens are often devised through trial-and-error in an attempt to treat observed symptoms. Unfortunately, underlying etiologies for most psychiatric disorders are currently unknown, so it is unclear how current treatments effect their biologic causes; as a result, unwanted side-effects are a common result (2). Computational Psychiatry research attempts to bridge the gaps in our understanding of the relationship between brain activity and human behavior, particularly in the context of psychiatric medicine, using computational tools including approaches that have long been developed in computational neuroscience basic research (3, 4). A particularly fruitful avenue of work has been the investigation of human decision-making and choice behavior where computational reinforcement learning (5, 6) has been a guiding conceptual framework.

Computational reinforcement learning (RL) theory (5, 6) describes a well-developed field of computer science research that has identified optimal algorithms by which theoretical “agents” may learn to make choices in order to optimize well defined objective functions. RL expanded into neuroscience with the discovery that RL concepts accurately model learning in mammalian brains (7–9). Specifically, temporal difference reward prediction errors (TD-RPE) have been shown to be encoded by dopamine neurons in the midbrains of non-human primates (7, 8) and rodents (9). The general idea of a “reward prediction error” calculation is “the reward received” vs. “the reward expected.” Neural and behavioral correlates to this general calculation can be found in nearly all experiments where expectations and deviations from these expectations can be controlled and robustly delivered to research subjects. However, some calculations of RPEs can explain more of the nuances in behavior as well as associated neural activity. For example, temporal difference reinforcement learning (TDRL) and the TD-RPE hypothesis of dopamine neuron activity can provide a mechanistic explanation of how an unconditioned stimulus (US) becomes associated with a conditioned stimulus (CS) in Pavlovian conditioning behavioral paradigms (Box 1); whereas Rescorla-Wagner based calculations of RPEs fail to provide this insight (12). Further, in quantitatively controlled experiments, the value of the RPEs can be designed to be significantly different depending on the manner in which the RPE is calculated.

Box 1 **Temporal difference reinforcement learning.**

Temporal difference reinforcement learning provides a computational framework to investigate how an agent might learn directly from experience. The goal of TDRL algorithms are to estimate the value of a particular state or of an action paired with a state (a state-action value) in order to maximize the rewards that can be obtained. At the core of this family of algorithms is a teaching signal called a temporal difference reward prediction error (TD-RPE), Equation 1:

(1) $$\delta_{t}=[outcome_{t}+\gamma V(S_{t+1})]-V(S_{t})$$

Here, δ_*t*_ is the TD-RPE quantity at time *t*, which is determined by evaluating the *outcome* magnitude (i.e., reward) observed at time *t* added to the expected future value, *V*(*S*_*t*+1_), of being in the present state. *V*(*S*_*t*+1_) is discounted by the term γ. All of this is compared to the overall value expected at time *V*(*S*_*t*_). This amounts generally to the idea of reward received vs. reward expected. However, the time indices cause the agent to evaluate not only what is received in the present state, but also consider how being in the present state increases or decreases the overall value of future states. It can be shown that the agent only needs to estimate one step into the future (5, 6) to have an optimal approach of associating the estimated value of future states with states that predict the occurrence of those future high (or low) value states. This reward prediction error (δ_*t*_) is then used to update the estimated value of the current state following Equation 2:

(2) $$\begin{array}{l} V^{\prime }\left(S_{t}\right)\leftarrow V\left(S_{t}\right)+\left(\alpha •\delta_{t}\right) \end{array}$$

where α is a fractional multiplier that controls how much the current reward prediction error updates the (apparently wrong) estimate of the value of the current state *V*(*S*_*t*_) to a new estimate $V^{\prime }\left(S_{t}\;\right)$.

**Figure 1:**
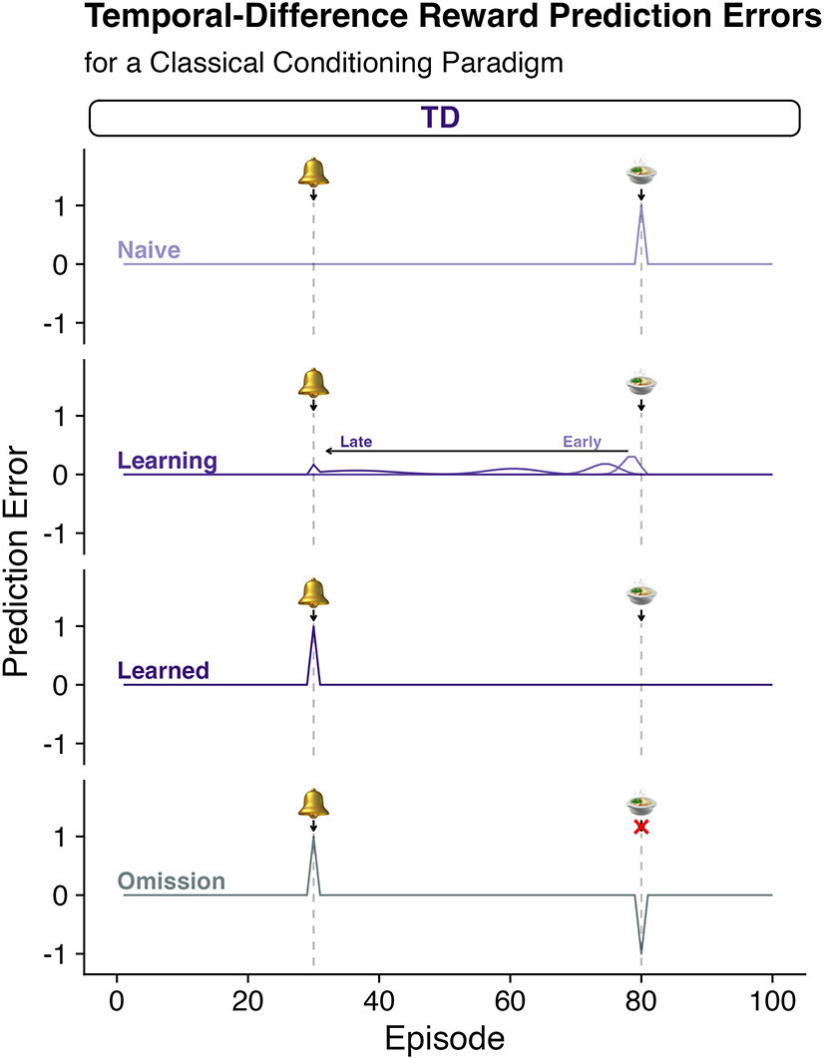
Pavlovian conditioning and temporal difference reinforcement leaning algorithms. Temporal Difference Reward Prediction errors (TD-RPEs) (see Box 1 for calculation) provide a teaching signal (Equation 1, Box 1) that updates estimates of the reward value associated with states of being or “episodes” (Equation 2, Box 1). Each row depicts the quantity of a TD-RPE predicted throughout various stages of learning. Row 1: “Naïve”; Row 2: as learning progresses from “Early” to “Late” stages, “Learning”; Row 3: after the agent has learned the association of the bell and the reward, “Learned”; and Row 4: after the association has been learned, but the anticipated reward is omitted, “Omission.” According to the TDRL algorithm, when a naïve agent encounters a surprising reward, a “better than expected” signal is generated (“Naïve”). This signal backs up in time as the teaching signal accounts for the collection of reward at the present state, but also the observation of being in a better than expected valued state given what is expected to happen one step (“*t* + *1”*) in the future. This causes the TD-RPE to back up in time to the state that is the earliest predictor of the subsequent reward. Over time the TD-RPE signal at the receipt of reward goes to zero as the expectations come to consistently match expectations. This also causes a negative reward prediction error when an expected reward is not delivered as shown in the “omission” trial.

Using these computations, one can simulate simple Pavlovian conditioning paradigms (Figure 1; or more complex operant behavior in humans). Figure 1 shows a simple CS-US pairing where the conditioning stimulus (CS) precedes a reliable reward (i.e., the unconditioned stimulus, US). Figure 1 shows that, prior to any learning, the surprising reward elicits a positive TD-RPE. Over time the learning rules expressed in equations 1 and 2 lead to the TD-RPE backing up in time (to earlier episodes of the trial) to the earliest reliable predictor: here, the bell. The lack of the response at *t* = 80 is consistent with there being a TD-RPE equal to zero – the reward received is exactly as expected. If, however, the reward is omitted (fourth row, “Omission”) a negative reward prediction error is emitted signaling that things are worse than expected.

Human decision-making can and has been successfully studied using this computational framework and state-action elaborations depicted with Q-learning. By interacting with the environment, we learn the consequences of our actions and adapt our behavior accordingly. These behavioral processes and TDRL models have been linked to fluctuations in the firing rates of mid-brain dopaminergic neurons, which have been shown to encode temporal difference (TD) reward prediction errors (RPEs; Equation 1) in response to better-than-expected or worse-than-expected outcomes (positive and negative RPEs, respectively) (10, 11). However, it remains unclear how these neurons can encode variations in the magnitude of punishment since negative RPEs (from simple omissions) drive the neurons to stop responding, which means that worse than expected outcomes that vary in magnitude (e.g., worse than simply not getting a reward) cannot be differentiated.

Recent work suggests that dopaminergic signals in the human brain also encode reward prediction errors (10, 11, 13), but whether these dopaminergic signals are specifically “temporal difference reward prediction errors” has not been demonstrated. Whether dopamine neurons encode temporal difference reward prediction errors vs. some other calculation of a “reward prediction error” signal is important because different specifications of the mathematical theory lead to different predictions about the relationship between the organism's (i.e., human) behavior and the underlying neural activity supporting it. Human behavior is not driven solely by the pursuit of reward and reward maximization. Negative feedback (e.g., injury) and the anticipation of negative outcomes (e.g., the threat of injury or death) have significant influence on the neurobehavioral processes underlying our behavior (14). Despite this, reward-focused TDRL-based accounts of the neural underpinnings of human decision-making and behavior have not explicitly addressed limitations in the ability of dopamine neuron encoding of TD-RPEs to account for and encode punishing experiences (15–18).

Temporal difference reinforcement learning and its use in computational neuroscience and computational psychiatry treats appetitive and aversive experiences as opposite ends of a unidimensional reward spectrum (5, 17, 18). In this way, aversive experiences are modeled mathematically in TDRL as a negatively signed “reward,” represented by a single scalar value. This traditional unidimensional representation of valence in TDRL theory stipulates that appetitive and aversive experiences are inherently anti-correlated in the natural environment and, further, predicts that rewards and punishments are processed and influence behavioral control processes symmetrically, putatively *via* the dopamine system. However, empirical and theoretical studies in psychology and neuroscience indicate that this assumption might not accurately reflect the nature of reward and punishment learning and associated behavioral responses in humans and other animals (15–23).

Genetic research into learning from feedback indicates that separate genes may govern the dopaminergic mechanisms underlying learning to positive and negative outcomes (24). Further, mammalian mesencephalic dopamine neurons demonstrate low baseline firing rates (25), and it is therefore unclear how pauses in dopamine neuron activity are able to effectively communicate magnitude variations across all aversive experiences (negative RPEs) in the same manner as for rewards (positive RPEs), or how downstream brain regions might decode this information conveyed by dopaminergic neuron silence and use it for further behavioral control (15). In primates, midbrain dopaminergic neurons demonstrate excitatory firing behavior following appetitive rewards and pauses following aversive punishments (7, 8, 26). However, evidence suggests that a separate population of dopaminergic neurons may demonstrate excitatory activity in response to punishing information (26–28). For example, different populations of dopaminergic neurons in the rodent ventral tegmental area (VTA) have been reported to respond with excitatory activity to rewarding or punishing outcomes, respectively (27). Local field potential (LFP) data in rodents corroborates this separation of dopaminergic excitatory activity for rewarding vs. aversive learning; namely, theta oscillations increase with rewarding—but not punishing—feedback (28). While these data support the idea that the “dopaminergic system” can encode both reward and punishment prediction errors, it also suggests that there are separate neural systems within the dopaminergic neuron pool that appear to partition along positively and negatively valenced feedback, which in turn may underlie dopamine's role in learning to escape aversive situations (29). That separate dopaminergic neurons (but also other neuromodulatory systems) may encode both reward or punishment related information independently complicates an otherwise simple explanation. However, the application of computational models may be one way that clarity can be gained as these models are explicit about how separate systems combine information and contribute to complex behaviors.

These and other insights have inspired new RL-based computational theories that aim to capture diverse aspects of the influence of rewards and punishments on human neural activity, choice behaviors, and affective experiences (18, 23, 29, 30). Still, the vast majority of RL computational models employed to study human neural systems and decision-making behaviors rely on a unidimensional representation of outcome valence as the modeled driver of adaptive learning. Though contemporary applications of TDRL theory to understanding human decision-making in psychiatric conditions have seen early successes, reward-centric computational theories are ultimately limited in their ability to distinguish the unique influence of rewards and punishments on human neural activity and associated affective behaviors that are manifest in a variety of psychiatric conditions.

Newer reinforcement learning models have incorporated novel reinforcement learning terms as a method to separately investigate behavior and neurochemical responses to appetitive and aversive stimuli (18, 24, 31). Collins and Frank conceptualize stimuli as evaluated by a “critic” encoded by phasic dopamine in the ventral striatum in Opponent actor learning (OpAL) (31). “Go” and “NoGo” weights are calculated separately for appetitive and aversive stimuli (31). These are hypothesized to be encoded by D1 receptors in the direct pathway, and D2 receptors in the indirect pathway, respectively (31). Another proposed way to decouple algorithmic representations of rewards and punishments is a valence-partitioned reinforcement learning (VPRL) proposed by Sands and Kishida (16–18). We use VPRL (Box 2) as one putative example for how models incorporating aversive feedback may differ from traditional RL models. VPRL is based on the observation that dopaminergic neurons have a low baseline firing rate (4 Hz), and may not have sufficient bandwidth to encode variations in punishment magnitude by decreases in firing (though see Bayer et al.) (17, 18, 25). VPRL provides a generative account that may explain asymmetric representations of positive and negative outcomes that guide adaptive decision-making. This includes the relative weighting of benefits vs. costs when making decisions. Further, it retains the successful accounting of TDRL-based optimal reward learning while hypothesizing a parallel independent process for punishment learning.

Box 2 **Valence-partitioned reinforcement learning.**

One hypothesized solution to the limitations of TDRL (Box 1) is to partition “outcomes” according to their valence. Positively valenced outcomes (e.g., those that promote survival and reproduction) are handled *via* a positive-valence system, whereas negatively valenced outcomes (e.g., those that would lead to death if unchecked) are handled by a negatively valenced system. Both systems are hypothesized, here, to learn optimally from experience and hence use the TDRL-prediction error, but for partitioned valence specific receptive fields (Equations 3 and 4):

(3) $$\delta_{t}^{P}=\{\begin{array}{c} outcome_{t}+\gamma^{P}V^{P}\left(S_{t+1}\right)-V^{P}\left(S_{t}\right)\;if\;outcome_{t}>0\\ 0+\gamma^{P}V^{P}\left(S_{t+1}\right)-V^{P}\left(S_{t}\right)\;if\;outcome_{t}\leq 0 \end{array}$$

(4) $$\delta_{t}^{N}=\{\begin{array}{c} \left|outcome_{t}\right|+\gamma^{N}V^{N}\left(S_{t+1}\right)-V^{N}\left(S_{t}\right)\;if\;outcome_{t}<0\\ 0+\gamma^{N}V^{N}\left(S_{t+1}\right)-V^{N}\left(S_{t}\right)\;if\;outcome_{t}\geq 0 \end{array}$$

$\delta_{t}^{P}$and $\delta_{t}^{N}$ are each TD-prediction errors that are calculated in much the same way as TD-RPEs in Box 1, except that the “outcome” processed at time t are conditioned on the positive (greater than zero, Equation 3) or negative (less than zero, Equation 4) valence. If the valence of the outcome is not within the respective positive or negative receptive field, then the outcome is treated as a null, or zero, outcome for that respective system.

As in TDRL, the prediction errors update representations of the appetitive (Positive system) and aversive (Negative system) values $V^{P}\left(S_{t}\right)$ (Equation 5) and $V^{N}\left(S_{t}\right)$ (Equation 6), respectively.

(5) $$\begin{array}{l} V^{P}\left(S_{t}\right)\leftarrow V^{P}\left(S_{t}\right)+\left(\alpha^{P}•\delta_{t}^{P}\right) \end{array}$$

(6) $$\begin{array}{l} V^{N}\left(S_{t}\right)\leftarrow V^{N}\left(S_{t}\right)+\left(\alpha^{N}•\delta_{t}^{N}\right) \end{array}$$

The estimated appetitive (positive system) and aversive (negative system) values $V^{P}\left(S_{t}\right)$ (Equation 5) and $V^{N}\left(S_{t}\right)$ (Equation 6) can then (at any time) be integrated to provide an overall estimate of expected value (Equation 7):

(7) $$\begin{array}{l} V\left(S_{t}\right)\leftarrow V^{P}\left(S_{t}\right)\;-\;V^{N}\left(S_{t}\right) \end{array}$$

**Figure 2:**
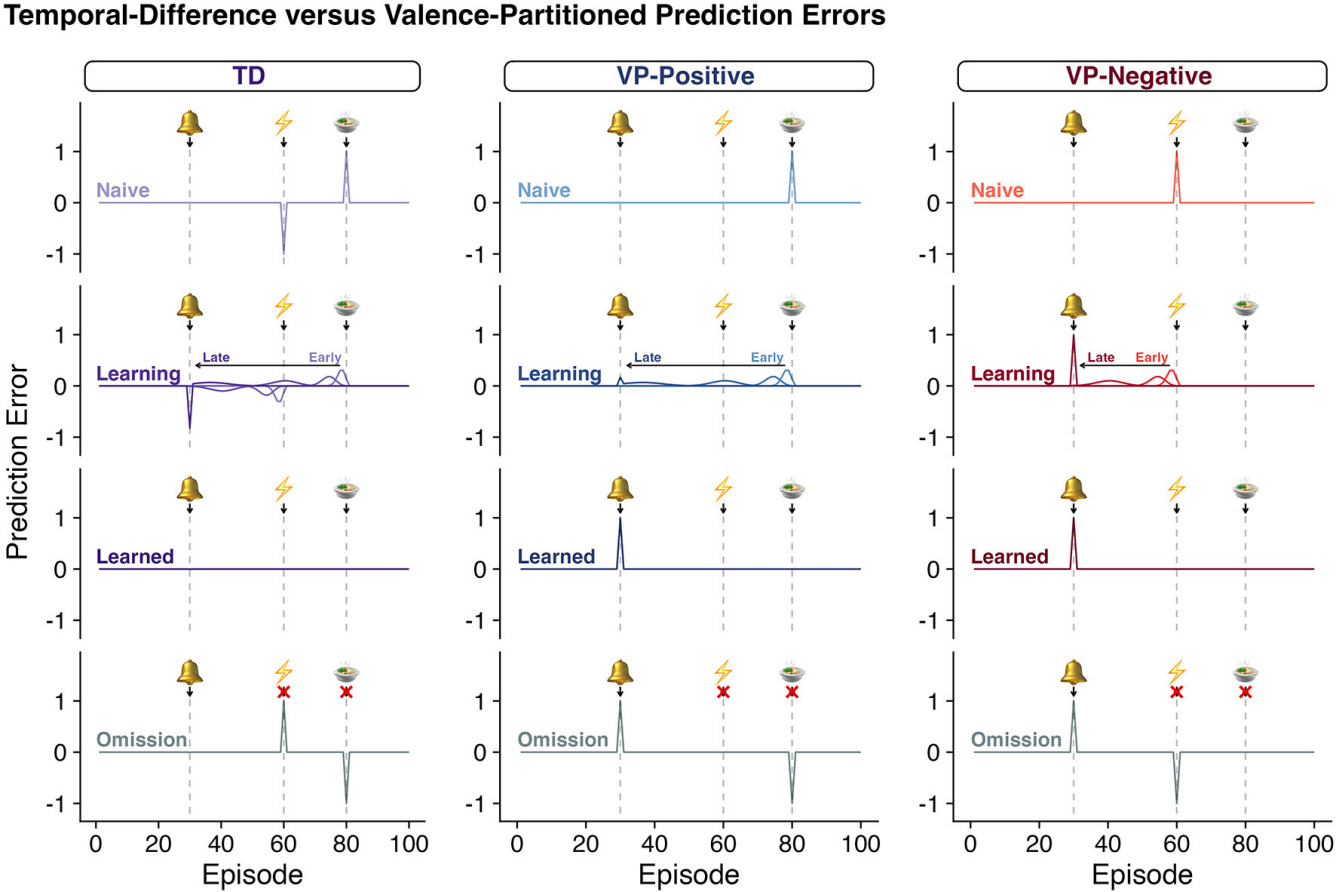
Classic TDRL algorithms do not distinguish rewards and punishments. As in Figure 1, each row depicts the quantity of a “prediction error” predicted throughout various stages of learning according to TDRL (column 1, Box 1, Equations 1–2) or valence-partitioned reinforcement learning (VPRL, columns 2 and 3, Box 2, Equations 3–7). Row 1: “Naïve”; Row 2: as learning progresses from “Early” to “Late” stages, “Learning”; Row 3: after the agent has learned the association of the bell and the outcomes, “Learned”; and Row 4: after the association has been learned, but the anticipated outcome are omitted, “Omission.” In a paradigm that contains both a reward and a punishment in each trial, classic-TDRL generates a positive RPE for surprising gains and a negative RPE for surprising losses (TD - “Naïve,” row 1). As these outcomes are learned, the TD-RPE backs up in time as specified in Eqs, 1, 2 and shown (TD - “Learning,” row 2; for rewards only in Figure 1). However, once these associations are learned (“Learned”, row 3) the positive and negative values of the eventual outcome cancel each other out and the associated TD-RPEs. This behavior of the TDRL algorithm is symptomatic of a system that does not independently distinguish rewards and punishments. As one hypothetical remedy, we propose VPRL (columns 2 and 3, Box 2, Equations 3–7). The prediction errors generated by the Positive System (VP-Positive) and Negative System (VP-Negative) behave in much the same way as the traditional TDRL algorithms, but by having these systems partitioned they are able to independently provide learning signals that can update both the positive and negative aspects of episodes and actions (if applied in Q-learning contexts). This would allow a system to use both error signals (predictive positive and predictive negative) to motivate behavior and evaluate the associated cost and benefits without unintentionally superimposing the valence of coincident events.

This approach maintains the optimal learning algorithm from TDRL reward processing and it is expected that the results of experiments that only vary reward will result in the same predictions when comparing traditional TDRL (Box 1) and this instantiation of VPRL (Figure 2). However, in experiments where punishment learning is investigated, the two approaches will have very different predictions about the calculated reward and punishment learning signals (Figure 2).

In this review, we discuss the benefits that reward-centric TDRL accounts have provided for computational psychiatric investigations into the brain-behavior based accounts of psychiatric conditions and highlight where considerations of punishment learning have also provided insight. We begin our discussion with two disorders that are classically hypothesized to have predominantly hyperdopaminergic action as a potential underlying etiology: addiction and schizophrenia (32, 33). In both behavioral and substance based additions, it is hypothesized that dopaminergic reinforcement systems are manipulated by repetitive risky decisions or pharmacological agents directly or indirectly (32, 33). In schizophrenia, hallmark positive symptoms like psychosis may result from hyperdopaminergic activity of D_2/3_ receptors in the striatum (34–36). However, application of a reinforcement learning framework demonstrates that hypodopaminergic action of stimuli may account for the duality of negative symptoms (37). We continue our discussion with the insights that computational reinforcement learning has had on understanding reward processing in disorders of primarily hypothesized hypodopaminergic signaling. Depressive symptoms found in major depressive disorder (MDD) may arise from altered reward and punishment learning, consistent with depression as a disorder of learning from feedback (38–40). We also highlight here that PTSD may be understood as a reinforcement learning disorder and discuss how future investigations using a computational framework may provide a roadmap for understanding the etiology of PTSD as well as the efficacy of current exposure-based therapies for PTSD.

## Behavioral addictions, substance use disorder, and impulse control disorder

Addiction has long been conceptualized as a spectrum disorder that includes substance based as well as behavioral addictions (41–43). The repetitive engagement in behaviors detrimental to an individual is required for the diagnosis of all addictions—including substance use disorders and behavioral addiction. Also, a loss of control or inhibition often defines the pathology of addictive disorders (1, 41–44). Work investigating the dopaminergic system and associated RL-models, in addiction, are largely focused on the behavioral reinforcing aspects of addictive stimuli, but what is largely lost in these depictions is the diminished influence of the punishing or aversive stimuli that are also experienced by the patient yet appear to have little influence on behavioral change. The following section will consider processes common to behavioral addictions and substance use disorders as part of an extended addiction spectrum and details relevant to specific disorders highlighted by name.

The initial stages of any addiction are suggestive of both classical and operant conditioning (45, 46). On first use, addictive substances or behaviors elicit physiological and psychological unconditioned responses, which increase the likelihood of repeating the actions preceding the drug-taking or behaviorally rewarding experience (47). TDRL prescribes an algorithmic approach for associating the unconditioned ‘reward' response with the conditioning stimuli or operant behavior (5, 6). Physiologically, these events are strongly associated with a sudden increase in extracellular dopamine levels and is likely associated with the psychological experience “better than expected” (11). In the TDRL framework, this reward prediction error and would serve as a teaching signal for updating the “value” of the preceding states and actions (5, 6). Importantly, the actual “value” of the psychological experience need not correspond to the “value” being updated in the dopaminergic-TDRL hypothesis (7, 8). Substances of abuse can directly and indirectly cause an increase in extracellular dopamine levels (32, 47, 48), which the brain may interpret as a signal to increase the estimated value of an on-drug state (32, 33). The hypothesis is that brain receives a signal that neurochemically reinforces the drug-taking state — akin to this state being of “higher value” than expected. This neurochemical reinforcement is not necessary associated with a positive subjective experience; in fact, individuals suffering from addiction may continue an addictive drug or behavior in an attempt to return to prior state (49–51). Notably, these repetitive behaviors in the face of negative feedback would appear as a relative underweighting of aversive or punishing feedback in addition to the relative overweighting of (no longer) rewarding feedback (49–51).

From this viewpoint, the pathology of addiction can be conceptualized as a disorder of learning caused by an over-valuation of behaviors associated with artificial increases in dopamine, but also an underweighting of consequent negative experiences. Dopamine increases are caused by pathological levels of a potentially addictive substance or behavior (51, 52). Such a connection between temporal difference reinforcement learning (TDRL) and addiction was put forth by Redish, who posited that aberrant RPE signaling in individuals suffering from substance use disorder (SUD) may lead to persistent use of addictive substances (32). As Redish noted, many drugs with addictive potential increase dopamine levels directly, like cocaine, or indirectly, like nicotine and heroin, and SUDs may be understood in the context of aberrant dopaminergic TDRL signaling (32). Indeed, individuals with SUDs have been observed to overvalue immediate, drug-associated stimuli and undervalue future alternative rewards (53, 54). This discounting of future outcomes is more severe in patients with SUDs including opioid-dependence and cocaine use disorder in studies assessing delay-discounting by survey tools and monetarily incentivized decision-making tasks (55, 56). Temporal discounting behavior has been shown to predict short-term relapse for individuals completing an inpatient detoxification program for cocaine, opioid, marijuana, and amphetamine addictions (57). Studies of methamphetamine use disorder also find these individuals to have altered learning with associated hyperactivation of striatal regions while performing reinforcement learning tasks in patients who have relapsed vs. those who have maintained abstinence (58, 59).

The conceptual link between reinforcement learning and addiction has been expanded to include the entire addiction spectrum, including behavioral addictions (BAs) (33). Individuals with BAs share patterns in cognition and decision-making with patients suffering from SUDs (60, 61). In light of these findings, gambling disorder was recategorized in the DSM-5 as the first non-substance, or behavioral, addiction (62). It is hypothesized that the same underlying dopaminergic pathways critical to reinforcement learning are altered in all forms of addictions (33). If this is true, then core characteristics in patients suffering along the spectrum of addictions (including substance use disorders) may be revealed by studying decision-making in specific behavioral addictions (1, 63). There is, however, a major difference between substance induced addictions and behavioral addictions — addictive substances can be neurotoxic at the levels typically consumed by individuals with substance use disorders, whereas behavioral addictions are not known to have similar neurotoxic or neurodegenerative profiles (1, 61, 63–65). Thus, the study of BAs may allow for a separation of the underlying neural mechanisms shared by all addictions without the confounding factor of the neurotoxicity-induced changes that are caused by specific addictive substances (i.e., cocaine vs. alcohol).

A special class of BAs, Impulse Control Disorder (ICD), has furthered the investigation of addiction and the role reinforcement learning mechanisms may play. Impulse control disorders (ICDs) are identified as rapid changes in risky behavior that arise secondary to dopaminergic therapies for the treatment of Parkinson's disease (PD), particularly dopamine receptor agonists (66). The current gold standard for assessing ICD history is the Questionnaire for Impulsive-Compulsive Disorders in Parkinson's Disease-Rating Scale (QUIP-RS), which is a self-report survey instrument (67, 68). In the QUIP-RS, ICD is defined as meeting a threshold of excessive behavior in one or a combination of the following categories: gambling, sex, buying, and eating (67, 68). These ICDs can be present as specific symptoms (for example, gambling or eating disorders) or as a combined group of ICD symptoms; the unifying factor is that the symptoms present as a rapid change in risky behavior elicited by dopaminergic action (67, 68). In a study of over 3,000 patients with PD, dopamine agonist therapy increased the odds of developing ICD by 2–3.5 times (69). Within the subgroup of patients with any ICD, over a quarter had two or more ICDs (69).

As ICDs occur naturally through the course of dopaminergic therapy for patients with Parkinson's disease, patients with ICD have been studied as a way to investigate the relationship between dopamine and behavioral addiction (66, 70, 71). Elevated impulsivity has been shown to be a hallmark both in patients with pathological gambling, an ICD, and patients with SUDs (72). [^11^C] raclopride positron emission tomography (PET) measurements during a gambling task have revealed increases in ventral striatal dopamine release in patients with Parkinson's disease with ICD compared to patients with Parkinson's disease without ICD (73).

A gap in the human addiction literature is a rigorous characterization of the individual neurochemical differences present in those who suffer from addiction and how those differences map to behavior. Future work using ICD as a model for behavioral addiction may be a promising avenue to uncover dopaminergic and behavioral signals with the potential to elucidate differences in reward and punishment processing and provide insight for therapeutic development. Dopamine increase during addictive drug use has been suggested to heighten learning following positive reinforcement, thus driving the transition between drug use and addiction and SUD (51, 71, 74). Similarly, it is possible that dopaminergic therapies used to treat PD may amplify dopaminergic signals involved in positive reinforcement, pushing those with an underlying predisposition for ICD into a symptomatic state. This may be particularly true for dopamine receptor agonists, where research suggests that these agents alter dopaminergic action specifically in the ventral and dorsal striatum (75). Directly investigating real-time dopamine signaling in response to reward and punishment in humans with and without a history of ICD is possible (11, 13, 76–78), and should provide critical insight into the neurochemical mechanisms underlying ICD and other addictive disorders.

Recent work suggests that punishment learning ought to also be more closely investigated in behavioral addiction and substance use disorder (79–82). In one study, pramipexole—a dopamine receptor agonist known to cause ICD—was given to individuals with SUD and found to increase learning from punishment (80). When patients with PD, with and without ICD, were presented with a probabilistic learning task including rewards and punishments, ICD patients on dopamine receptor agonists demonstrated a relatively lower learning rate to negative reward prediction errors (83, 84). Individuals with opioid use disorder completed the same task, but the researchers concluded that individual variability in learning to both rewards and punishments did not allow for detection of differences across individuals with and without SUD (84, 85). A separate study investigating heroin dependency found that individuals with a heroin dependency learned to successfully acquire rewards with equal accuracy as a control group, but the control group outperformed the group with heroin dependency when challenged to avoid punishments (86).

Further research on punishment learning in addiction may expand treatment options for addictive disorders, even employing medications already on the market for alternative uses (87). For example, the antipsychotic sulpiride, a D_2_ receptor antagonist, has already demonstrated differential action on reversal learning to rewards and punishments in gambling disorder vs. a control group (87). These results suggest that further research targeting investigation of punishment may reveal translational potential within differences in decision-making to punishing outcomes in ICD and across the addiction spectrum (81, 82).

## Schizophrenia

Schizophrenia is a psychiatric disorder diagnosed based on a spectrum of positive and negative symptoms (1, 88, 89) (Supplementary Table 1). Positive symptoms are often conceptualized as the outwards presentation of the disorder, notably hallucinations, delusions, and disordered speech (1). Negative symptoms include the inwards presentation of the disorder, which may consist of diminished emotional expression, asociality, anhedonia, avolition, and alogia (1). The dopamine hypothesis of schizophrenia suggests that dopamine dysregulation explains the characteristic signs and symptoms of the disorder (37, 89, 90). Recent animal and neurochemical imaging research — including positron emission topography (PET) and single-photon emission computed topography (SPECT) — refined the dopamine hypothesis to specifically include elevated presynaptic dopamine in the striatum as a key factor underlying schizophrenia (91). This hypothesis predicts that the divergent symptomology experienced in patients with schizophrenia (Sz) may result from altered dopaminergic receptor activity affecting distinct brain regions. Evidence from additional PET and SPECT imaging experiments shows that positive symptoms result from hyperdopaminergic activity of D_2/3_ receptors in the striatum (34–36). Negative symptoms and cognitive impairments may be a result of hypo-frontality, or decreased D_1_ receptor density in the prefrontal cortex (89, 91, 92). Alternatively, schizophrenia may be caused by increased spontaneous dopamine release punctuated by relatively decreased phasic responses to reinforcing stimuli (37). Based on this evidence, current research aims to determine how cognitive and behavioral processes regulated by striatal dopamine and dopaminergic projections to cortical regions are altered in Sz.

Cognitive impairments may be the sentinel sign of Sz—often appearing prior to positive symptoms classically associated with the disorder (93). Set-shifting in particular is a potential cognitive impairment in Sz with applicability to the reinforcement learning field (94–96). Set-shifting tasks require participants to ‘shift' their attention across different goals based on feedback; for example, the Wisconsin Card Sorting Test (WSCT) requires participants to sort cards based on rules that change throughout the test (96–98). It is acknowledged that set-shifting may measure a variety of cognitive skills, including working memory (96). Working memory is an important intermediate for RL because it is essential for integrating relevant information during goal-directed behavior (99, 100). Anatomically, working memory is modulated by dopamine in the prefrontal cortex and striatum. PET imaging and behavioral studies show that dopamine levels and working memory are related in a U-shaped fashion, with both excess and insufficient levels of dopamine in the prefrontal cortex and striatum impairing working memory (101–105). In Sz, these alterations to dopamine function impair RL processes leading to inaccurate value calculation and non-optimal weighing of potential outcomes (106). Therefore, patients with Sz who possess more intact working memory make more optimal decisions, whereas those with alterations to their working memory make less optimal decisions (106).

In addition to weighing potential outcomes, working memory is associated with alterations in delay discounting of rewards in Sz (101). Delay discounting is the tendency to discount future rewards as a function of time (i.e. the subjective value of a reward declines as the time to the reward increases) (101, 107). Patients with Sz have been shown to have greater delay discounting of rewards, meaning they prefer smaller immediate rewards rather than larger later rewards (101). While other cognitive processes have been hypothesized, it is believed that impaired working memory contributes to greater delay discounting (101, 102, 108). These findings suggest that greater delay discounting may add noise to value estimation functions resulting in altered reward value calculations in Sz (108). Thus, patients with Sz who exhibit impaired working memory, demonstrate greater delay discounting, which results in abnormal reward learning (108).

A hypothesis of shared striatal circuity that modulates both reinforcement learning (RL) and the dopaminergic dysfunction in Sz has led research into the relationship between schizophrenia and altered adaptive decision-making behavior. Findings based on PET and SPECT research, human neuroimaging studies, and computational models suggest that RPE signaling in the striatum is impaired in Sz compared to controls (91, 106, 109–111). Differences in RL are specifically evident in tasks where patients with Sz are required to utilize positive feedback to optimize rewarding outcomes, resulting in abnormal RPE signaling and altered activity in the ventral striatum (106, 109–111). This abnormal RPE signaling and altered striatal activation is thought to present as behavioral differences in reward and punishment learning in Sz. Reward learning in Sz differs based on the patient's current experience of positive or negative symptoms. When positive symptoms are experienced, patients with Sz exhibited alterations in reward learning specifically as a rigidness to maintain certain cue-reward associations, even when it was evident that this association was no longer rewarding (106). This association between reward learning and positive symptoms is exemplified during a probabilistic reward and punishment task, in which patients with Sz performed similar to patients with bipolar disorder with psychotic features, both disorders sharing psychosis as a potential symptom (109). Recent work has attempted to model choice proportions and reaction-time simultaneously using a reinforcement learning drift diffusion model (RLDDM) (112). A RLDDM was able to mathematically explain both choice proportions and reaction time performance during reward learning for those with Sz, and a trend for significant group differences in learning rate was found when comparing those diagnosed with Sz to healthy controls (112).

Patients with Sz who display negative symptoms show alterations in reward learning that lead to a tendency to fail to appropriately estimate and use future rewards to guide decision-making behavior (110, 111). This means patients experiencing negative symptoms tend to struggle to rapidly learn relative reward value and they tend to favor the immediate preceding reward choice, even if that option is less likely to be rewarding in the future (110, 111). Therefore, patients with Sz experiencing negative symptoms tend to select the option with the greatest immediate reward even if longer term rewards will be greater (110, 111). The alteration in reward learning to select and stay with immediate rewarding outcomes is significantly correlated with avolition, defined as the lack of motivation or ability to initiate or persist in goal-directed behaviors (110). Using a RL framework to model these differential associations and alterations in reward learning may provide additional understanding of the mechanisms underlying both positive and negative symptoms in Sz.

In addition to reward learning, patients with Sz show differences in punishment learning, though results in the literature are mixed. For example, patients with Sz have been shown to maintain sensitivity to rewarding stimuli but undervalue potential losses (108). Another study reported no differences in punishment learning on a probabilistic punishment task between patients with Sz and control individuals (109). However, other work found a reduction in the use of punishments to guide future choice behavior in patients with Sz (98, 113, 114). These conflicting results may be clarified by an approach using RL that explicitly accounts for the valence differential of both punishment learning and reward learning. A potential approach that explicitly accounts for a punishment learning system that is distinct from the reward learning system would allow one to experimentally and computationally control different aspects of reward and punishment to determine whether one or the other or both are altered in patients with complex presentations of positive and negative systems (18).

Schizophrenia is a severe psychiatric disorder characterized by alterations in dopaminergic activity eventuating in positive, negative, and cognitive symptoms (115). Alterations in dopamine receptor activity are known to be critical for differences in reinforcement learning behavior in Sz. Changes in RL in Sz appear to be contingent on one's current experience with positive or negative symptoms, which result in differences in working memory, delay discounting, and value calculation (101, 102, 106, 108). These neural and behavioral changes are thought to underlie aberrant decision-making in Sz (101, 102, 106, 108, 115). Using novel computational RL models that explicitly express the role of various factors could provide clarification of conflicting results and further the understanding of the divergent symptomology experienced by patients with Sz.

## Depression

Major depressive disorder is a leading cause of global disability, affecting over 300 million people worldwide (116). Despite this prevalence ~50% of individuals with MDD do not fully recover using traditional interventions, signifying the need for a better understanding of the neural mechanisms underlying depressive behaviors (117). Though depressed mood is standard for MDD diagnosis, symptoms that meet the DSM-5 criteria vary and may include differences in cognitive function and decision-making (118–120). Emerging evidence suggests these differences may arise from altered reward and punishment learning, characterizing depression as a reinforcement learning disorder (38–40). These behavioral differences are shown to correlate with altered brain activity in a myriad of regions responsive to rewarding and punishing feedback (for example, the prefrontal cortex, nucleus accumbens, amygdala, habenula, anterior insula, or orbitofrontal cortex) (121–125). Reinforcement learning models provide objective characterizations of dynamic brain activity and behavior that are lacking in clinical assessments and may lead to a better understanding of how various brain regions and their interactions are functionally and structurally altered in depression (126). However, the ability of these models to accurately represent the neural processes underlying reward and punishment learning will determine their utility in advancing our understanding and treatment of depression.

Temporal difference reinforcement learning models (5, 6) of human choice behavior are often used to study psychiatric populations including patients with depression. These standard algorithms are grounded in the RPE hypothesis that states that dopaminergic neurons encode the difference between an expected and experienced outcome. The role of reward has been well studied in the context of TDRL models of dopaminergic activity (7, 8, 17). TDRL models are frequently paired with decision-making tasks and functional magnetic resonance imaging (fMRI) to examine choice behavior and associated changes in neural response as humans adaptively learn from rewards and punishments (22, 127–129). This methodology is foundational to our present understanding of reward learning differences specific to depression, whereby many studies support diminished RPE signaling in response to rewarding outcomes, such as monetary rewards, in unmedicated patients with MDD (121). This diminished neural activity is shown within cortico-striatal circuits including the ventral tegmental area (VTA), nucleus accumbens, ventral striatum, amygdala, hippocampus, and prefrontal cortex (121, 122, 130, 131). Diminished functional connectivity during reward learning is further observed in unmedicated patients with MDD between regions such as the VTA and striatum and between the nucleus accumbens and midcingulate cortex (132). Collectively, studies assessing these reward-learning circuits demonstrate that strength of connectivity correlates negatively with both number of depressive episodes and depression severity (130, 132–135). It is hypothesized that this weakened RPE signaling in depression stems from dopamine signaling deficits downstream of the VTA, which is posited to generate RPE signals (136, 137). For example, one study found intact striatal RPE signaling in patients with MDD during a non-learning task (138). Other studies support diminished RPE signaling in this same brain region in patients with depression during reward learning (99, 130, 131). Together, these findings suggest that reward learning impairments in depression may stem from deficits in RPE signaling rather than a fundamental failure of RPE computation.

Multiple treatment modalities are suggested to improve reward learning impairments experienced by patients with MDD, including antidepressant medications, psychotherapies, and brain stimulation techniques (100, 113, 139). The mechanisms of action for these treatments are diverse, but evidence points (overall) to a role that allows an increase in the representation of RPEs that are generally hypothesized to be encoded by phasic dopamine. Antidepressants are pharmacological agents that modulate serotonin, norepinephrine, or dopamine concentrations in the brain (140). One study found that patients with MDD receiving long-term citalopram, a selective serotonin reuptake inhibitor (SSRI), show increased RPE signaling in the VTA (141). In contrast, diminished VTA RPE signaling was observed among unmedicated patients with MDD and in controls after receiving a 20 mg dosage of citalopram for only 3 days (141). Electrophysiological evidence suggests that SSRIs initially inhibit spontaneously active VTA dopamine neurons through 5-HT 2B/2C receptor pathways (142, 143). However, after long-term administration, these receptors become hyposensitive while DA D2/D3 receptors have been shown to increase (142, 143). The resultant rise in spontaneously active VTA dopamine neurons is hypothesized to drive the enhanced RPE signaling shown in antidepressant-responsive patients (141). The increased responsiveness of DA D2 and D3 receptors are similarly seen in the other types of antidepressants to increase mesolimbic dopaminergic function over time (144). Numerous other studies indicate normalized neural response to RPEs in regions such as the ventral striatum, ventrolateral prefrontal cortex, and orbitofrontal cortex after antidepressant treatment, suggesting the long-term action of these medications may be to modulate RPE signaling and reward learning (145, 146). Further, these findings are consistent with a tight interaction between opponent serotonin and dopamine neural systems generally, but also in the underlying pathology of MDD (15, 147).

Depression is also treated with cognitive behavioral therapy (CBT), an efficacious psychotherapy theorized to reduce symptoms in part through changing learning (114). A recent study found that after 12 weeks of CBT, normalization of reward learning rate occurred in patients with depression alongside improved anhedonia and negative affect (148). Another fMRI study demonstrates that increased neural activity in the ventral striatum, which is known to contain dense regions of dopaminergic terminals, correlates with increased RPE signaling and response to CBT (149). In addition, transcranial magnetic stimulation (TMS) is a common noninvasive brain stimulation treatment that delivers magnetic pulses to regulate the activity of cortical and subcortical structures (150). One study reported increased functional connectivity between the VTA, striatum, and prefrontal cortex among patients with depression responsive to TMS compared with those unresponsive to the procedure (151). Overall, these studies support improved reward learning among patients with depression responsive to various treatment modalities. These behavioral results suggest that prolonged treatments induce a series of adaptive signaling changes downstream of respective therapeutic targets which, in effect, improves reward learning and neural plasticity. It is not clear what mechanism allows the observed increases in reward learning rates following these brain stimulation treatments; however, a positive change in the ‘reward learning rate' parameter in TDRL models is consistent with the hypothesis that RPEs (hypothesized to be encoded by phasic changes in dopamine) gain influence in effecting behavioral change after these treatments.

Certain brain regions are posited to integrate reward and punishment information encoded by separate, opponent, neural systems relevant to depression. This opponent systems theory hypothesizes that neural systems that track negative reward prediction errors may include serotoninergic or a subset of dopaminergic neurons distinct from those that encode RPEs (16, 17, 23). Prediction-error-driven learning is also associated with subjective feelings, a critical aspect of depression as a disease of suffering. ‘Better or worse than expected' events drive learning and changes in behavior but also are hypothesized to drive dopaminergic signals and thereby affect momentary mood in response to rewarding outcomes (152, 153). One study implemented a “happiness” model using trial-level choice parameters including the value of certain rewards, the expected value of a chosen gamble, and associated RPEs to predict subjective ratings of happiness (138). Results showed that baseline mood parameters correlate with depressive symptoms, though there was unexpectedly no difference between patients with MDD and controls when assessing the expression of dopaminergic RPEs and their impact on self-reported happiness (138).

There has also been investigation into the structural abnormalities associated with reward and punishment learning systems in depression. These structural foundations for which functional (reward and punishment) prediction error signals are carried are an important aspect to consider as decreases in the integrity of physical connections within reward and punishment valuation networks may underlie loss of reward and punishment efficacy in depression (154). At this time, results appear inconsistent across studies, with disagreement in precisely where the changes exist with respect to these pathways. The major reward system pathways include the cingulum bundle (CB; ventromedial frontal cortex to posterior parietal and temporal cortices), uncinate fasciculus (UF; ventromedial frontal cortex to amygdala), and superolateral medial forebrain bundle (slMFB; anterior limb of internal capsule to frontal brain regions) (155–157). One of the main indices derived using diffusion tensor imaging (DTI) is fractional anisotropy (FA), which is a measure of water movement. FA can provide information about the structural integrity and axonal properties within white matter tracts, as healthier, more myelinated or organized tracts will restrict movement to one direction along the axons. Several DTI studies have shown decreased FA, indicating diminished white matter integrity, in various areas within the CB of patients with MDD (158–161). However, other DTI studies have found no changes in FA of the CB (162–164). These inconsistencies may be due to several factors, such as the effects of medication and disease history that differ between studies (155). The microstructure of the CB may also be indicative of vulnerability to depression, as suggested by family history studies, making it a target for study in depression identification and diagnosis (165, 166). Within the UF, most studies agree that FA is decreased in adult patients with MDD (164, 167, 168). However, results are less consistent in adolescents, with some studies reporting increases in FA while others report decreases, indicating that age and development may be a determining factor (162, 163, 169). Microstructural alterations have also been shown within the slMFB in patients with acute and treatment-resistant depression, making it a target for therapies such as deep brain stimulation to alleviate depression symptoms (170–175). However, unlike CB abnormalities, changes in the UF and slMFB have yet to provide evidence of a biomarker for depression in those with familial history. Interestingly, several studies show that in these pathways of the reward circuit, decreased FA is associated with higher overall melancholic symptoms and depression severity as determined by depression rating scales (155, 166, 170).

Current TDRL research investigating depression also suggests altered punishment learning within certain brain regions (for example, the habenula, insula, or medial prefrontal cortex) (123, 124). The lack of uniform incorporation of punishment learning in dopamine-TDRL theory may have produced conflicting results in the literature regarding differences in punishment learning reported for patients with and without depression (125, 176, 177). For example, fMRI studies show increased “negative-reward-prediction-error”-associated neural responses to monetary loss in the habenula, anterior insula, and lateral orbitofrontal cortex in unmedicated patients with MDD (123, 134). Further, negative prediction error signals in the habenula are shown to correlate positively with number of depressive episodes, suggesting punishment-related habenula activation increases with disease burden (130, 178). Alternatively, unmedicated patients with MDD have also shown decreased negative prediction error response to monetary loss in the insula, habenula, and prefrontal cortex (177). Other studies show no group differences in habenula activation or connectivity strength between the VTA and habenula for unmedicated patients with MDD and healthy controls during gain or loss conditions (130). Importantly, these studies use similar monetarily-incentivized reinforcement learning tasks yet report different directionality of negative-reward-prediction-error-responsive neural activity. There are also reports of overlapping positive reward prediction error and negative reward prediction error signaling (179); for example, the anterior cingulate cortex shows increased reward prediction error and negative reward prediction error activity among individuals genetically at-risk for depression, and the insula and VTA show increased reward prediction error and negative reward prediction error activity among unmedicated patients with MDD (180).

There has been much less focus linking punishment learning and white matter differences associated with depression. One study did report a significant decrease in mean FA within the VTA-lOFC (ventral tegmental area — lateral orbitofrontal cortex) connection tract of the MFB associated with punishment processing in depressed individuals compared to non-depressed controls (170). However, these results were significant only between controls and patients suffering from melancholic MDD and did not remain significant between controls and non-melancholic MDD. As such, white matter structural abnormalities may be a key feature in the reward and punishment dysfunction associated with depression symptoms and severity.

Overall, reward learning has been widely investigated in depression, but future research may use computational frameworks to explicitly parse reward and punishment learning processes to identify and delineate hypothesized reward and punishment learning neural circuits. In turn, this may help pinpoint reward and punishment system pathways in the human brain and better capture how humans—and especially patients with MDD—may react differently from complicated interactions of appetitive and aversive outcomes in natural experience. While computational formulations that better account for these systems will help, it is important to note that factors such as clinical heterogeneity, different subtypes of depression, and variability in study methodology likely also contribute to discrepancies in the current literature. Still, more detailed computational characterizations of patient behavior, charateristics, and patient populations may provide an approach to better stratify patient subtypes for more effective targeting of individualized therapeutic strategies. The precision gained through a computational lens may provide hypotheses about how distinct learning processes and associated circuits are altered in depression. These data, paired with white matter abnormalities in individual patients with depression, including MDD, may provide important behavioral, functional, and structural measures to advance our current understanding and treatment of depression.

## Post-traumatic stress disorder

A diagnosis of post-traumatic stress disorder (PTSD) by DSM-5 criteria must involve exposure to actual or threatened death, serious injury, or sexual violence; after such an event, the following must be present: (1) the presence of intrusion symptoms associated with the traumatic event; (2) persistent avoidance of stimuli associated with the traumatic event; (3) negative alterations in cognition or mood beginning or worsening after event; (4) marked alteration in arousal and reactivity associated with the traumatic event; and (5) symptoms lasting longer than a month (1, 181). Though the exact mechanism is not yet understood, PTSD has been described as a reinforcement learning deficit (182, 183). Patients who suffer from PTSD have come to associate punishment with environments which are normally neutral or even typically rewarding (1, 181). Psychologically, patients act as though they predict and relive traumatic experiences in relatively innocuous settings (1, 181). Researchers have taken first steps toward understanding reinforcement learning mechanisms underlying PTSD through work developing our understanding of safety cues (182, 183).

It is known that PTSD patients are unable to inhibit fear response in the presence of environmental cues that indicate that an environment associated with punishment will not result in punishment (184). For example, a combat veteran may experience uncontrollable fear in response to a previously learned aversive stimuli (e.g., helicopter sound) when surrounded by many other cues that signal safety (e.g., company of a loved one in a non-threatening environment) (184). Inhibition of fear potentiated startle phenomena in the presence of a safety cue was first observed in a control human cohort in 2005 (185). In a later study, inhibition of fear-potentiated startle to a safety cue was associated with PTSD symptom severity (186).

In humans the safety signal phenotype of PTSD is seen in the startle response, where startle is attenuated by the presence of the safety signal. Startle responses of PTSD patients are not attenuated by the presence of a safety signal compared to controls (187). Administration of L-Dopa during safety learning results in safety memories which are context independent (188). Enhanced dopaminergic activity following omission of aversive stimuli as seen in dopamine rebound is one endogenous mechanism which could mimic this experimental observation. Further research is necessary to determine if physiologic dopamine rebound is related to safety learning in PTSD. Modeling dopamine rebound and safety learning in a reinforcement learning framework offers a computational approach to address the relationship between PTSD and impaired safety learning. Future work may utilize computational reinforcement learning algorithms to express explicit hypotheses about the way reward and punishment systems interact such that the mechanisms underlying PTSD may be disentangled and new therapeutics developed.

## Conclusions and future directions

Temporal difference reinforcement learning theory and the calculation of reward prediction errors therein has been instrumental in providing insight into the information that dopamine neurons encode. The idea that dopamine neurons encode “reward prediction errors” that are important for updating expectations and guiding choice behavior has been a critical one for understanding motivated mammalian behavior and has led computational psychiatric investigations into a number of psychiatric conditions where subjective suffering and aberrant decision-making are hallmark features.

The gains that have been made using TDRL, and computational reinforcement learning theory more generally, to guide computational psychiatric investigations are undeniable. Future computational psychiatric investigations may utilize VPRL as it is described here or some other explicit approach to hypothesize about how the brain may learn to adapt in the face of punishing outcomes. The best approach is to be determined, but we hope to have made the case that some way of accounting for this fundamental aspect of human behavior and psychiatric illness ought be accounted for and investigated further.

We hope to provide those interested in computational psychiatry a clear picture of the importance of extending the widely successfully computational reinforcement learning framework to include punishment learning. The mathematical explication of reward learning mechanisms has allowed rigorous testing of hypothesized processes including the role dopamine neuron activity and dopamine release in the brain may play in human cognition generally, but specifically how these processes may be altered in psychiatric illness (81). We hope to encourage investigators in the area to consider the potential impact equally explicit mathematical representations of punishment learning may have, especially since this is a major gap in the current state of mental health research (189).

## Author contributions

BL and KK conceived of and planned the layout and content of this review. BL, RJ, ED, JT, JH, LS, KS, CJ, EF, AJ, and KK contributed to the literature review and drafting of individual sections of this work. All authors reviewed and edited this manuscript in its entirety for intellectual content, provided final approval of the version to be submitted for review and publication, and agree to be accountable for all aspects of the work including ensuring that questions related to the accuracy or integrity of any part of the work are appropriately investigated and resolved.

## Funding

The authors would like to acknowledge the following funding sources that supported this work: National Institutes of Mental Health (KK: R01MH121099 and R01MH124115), National Institutes of Drug Abuse (KK: R01DA048096 and P50DA006634, BL: F30DA053176, and LS: F31DA053174 and T32DA041349), the National Center for Advancing Translational Sciences (KK: UL1TR001420 and KL2TR001421), and KS is funded by T32NS115704 from National Institute of Neurological Disorders and Stroke and National Institutes of Mental Health (The program, JSPTPN, is jointly sponsored by the NINDS and NIMH - https://www.ninds.nih.gov/funding/training-career-development/institutional-grants/jointly-sponsored-institutional-predoctoral-training-program-jsptpn).

## Conflict of interest

The authors declare that the research was conducted in the absence of any commercial or financial relationships that could be construed as a potential conflict of interest.

## Publisher's note

All claims expressed in this article are solely those of the authors and do not necessarily represent those of their affiliated organizations, or those of the publisher, the editors and the reviewers. Any product that may be evaluated in this article, or claim that may be made by its manufacturer, is not guaranteed or endorsed by the publisher.
