# Supplementary figures and images for: Computational reinforcement learning, reward (and punishment), and dopamine in psychiatric disorders
Source: Front Psychiatry. 2022 Oct 20;13:886297. doi: 10.3389/fpsyt.2022.886297 (PMC9630918; doi:10.3389/fpsyt.2022.886297)

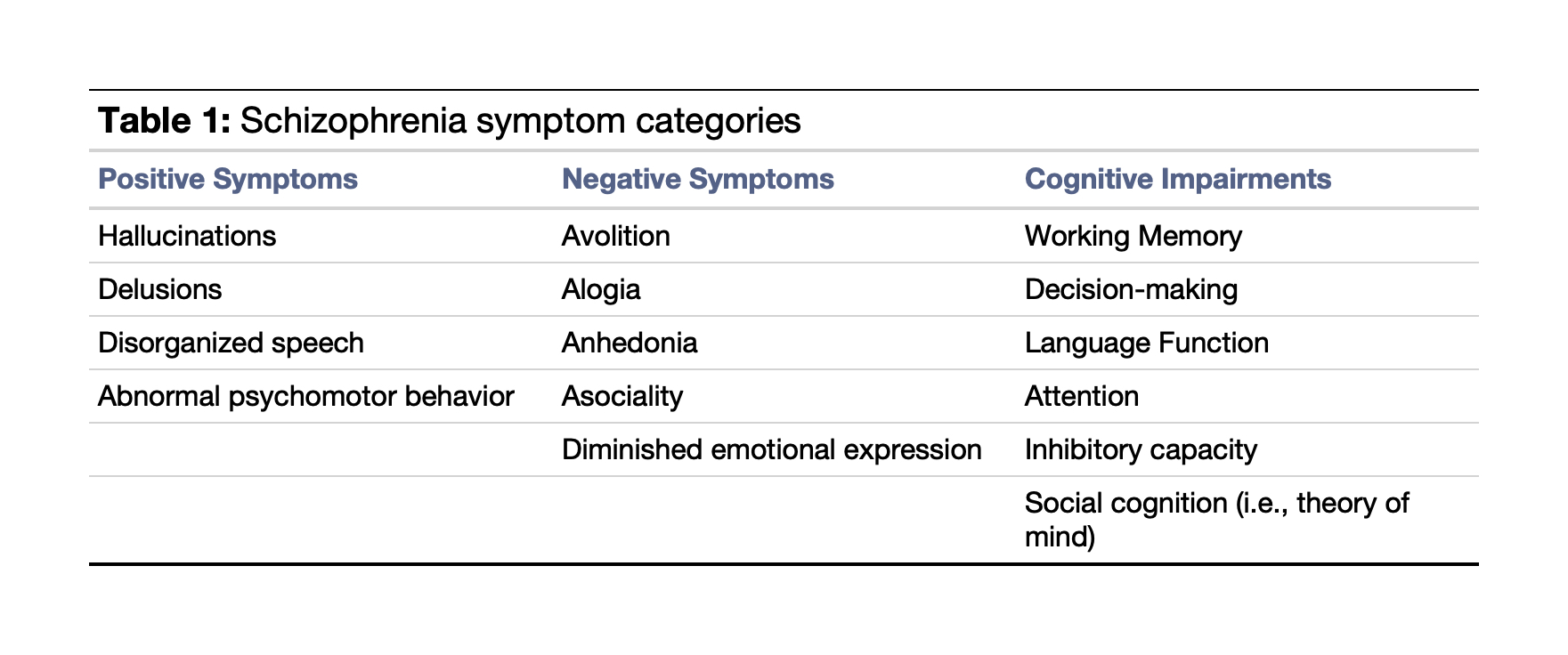

Supplement: Supplementary file 1 [file Image_1.JPEG]
